# UPCLASS: a deep learning-based classifier for UniProtKB entry publications

**DOI:** 10.1093/database/baaa026

**Published:** 2020-05-04

**Authors:** Douglas Teodoro, Julien Knafou, Nona Naderi, Emilie Pasche, Julien Gobeill, Cecilia N Arighi, Patrick Ruch

**Affiliations:** 1 Geneva School of Business Administration, CH-1227, University of Applied Sciences and Arts Western Switzerland, HES-SO, Geneva, Switzerland; 2 Text Mining Group, Rue Michel-Servet 1, CH-1206, SIB Swiss Institute of Bioinformatics, Geneva, Switzerland; 3 Center of Bioinformatics and Computational Biology, 15 Innovation Way, 19711, Department of Computer and Information Sciences, University of Delaware, Newark, DE, USA

## Abstract

In the UniProt Knowledgebase (UniProtKB), publications providing evidence for a specific protein annotation entry are organized across different categories, such as function, interaction and expression, based on the type of data they contain. To provide a systematic way of categorizing computationally mapped bibliographies in UniProt, we investigate a convolutional neural network (CNN) model to classify publications with accession annotations according to UniProtKB categories. The main challenge of categorizing publications at the accession annotation level is that the same publication can be annotated with multiple proteins and thus be associated with different category sets according to the evidence provided for the protein. We propose a model that divides the document into parts containing and not containing evidence for the protein annotation. Then, we use these parts to create different feature sets for each accession and feed them to separate layers of the network. The CNN model achieved a micro F1-score of 0.72 and a macro F1-score of 0.62, outperforming baseline models based on logistic regression and support vector machine by up to 22 and 18 percentage points, respectively. We believe that such an approach could be used to systematically categorize the computationally mapped bibliography in UniProtKB, which represents a significant set of the publications, and help curators to decide whether a publication is relevant for further curation for a protein accession.

**Database URL:** https://goldorak.hesge.ch/bioexpclass/upclass/.

## Introduction

Due to the deluge of research data created at ever-increasing rates, the scientific community is shifting towards and relying on curated resources [1]. Biocurated resources provide scientists with structured, computable-form knowledge bases extracted from unstructured biological data, particularly published manuscripts, but also other sources, such as experimental data sets and unpublished data analysis results [2]. The UniProt Knowledgebase (UniProtKB) aims to collect functional information on proteins with accurate, consistent and rich annotation. In addition to capturing the amino acid sequence, protein name, taxonomic data and citation information, it includes literature-based information about different topics, such as function and subcellular location. UniProtKB combines reviewed UniProtKB/Swiss-Prot entries, to which data have been added by expert biocurators, with unreviewed UniProtKB/TrEMBL entries, which are annotated by automated systems, including rule-based [3]. The reviewed section represents <1% of the knowledgebase. Biocurators select a subset of the available literature for a given protein, representing the landscape of knowledge at a given time [4].

Given the extent of the datasets processed by biocurators, commonly in the range of thousands to millions of publications, the scalability of biocuration in life sciences has often been scrutinized [5]. Text mining [6] has been proposed as one solution to scale up literature-based curation, especially to assist biocuration tasks via information retrieval, document triage, named entity recognition (NER) and relation extraction (RE) and resource categorization [7, 8, 9, 10]. Textpresso Central, for instance, provides a curation framework powered with natural language processing (NLP) to support curators in search and annotation tasks in WormBase and other databases [11]. Similarly, BioReader focuses on the classification of candidate articles for triage [12]. Providing positive and negative examples, the framework is able to automatically select the best classifier among a range of classification algorithms, including support vector machine (SVM), *k*-nearest neighbours and decision tree. Tagtog leverages manual user annotation in combination with automatic machine-learned annotation to provide accurate identification of gene symbols and gene names in FlyBase [13]. Text mining has also supported more specific curation tasks, such as protein localization. LocText, for example, implements a NER and RE for proteins based on SVM, achieving 86% precision (56% F1-score) [14]. To address the common issue of class imbalance in biocuration, an ensemble of SVM classifiers along with random under-sampling were proposed for automatically identifying relevant papers for curation in the Gene Expression Database [15].

More recently, with the success of deep learning in image and text processing applications [16], deep learning models have been increasingly applied to biocuration. Deep learning classification and prediction models for text—the main use-cases in biocuration—are heavily supported by neural language models, such as word2vec [17] and Global Vectors (GloVe) [18], and lately by Embeddings from Language Models (ELMo) [19] and Bidirectional Encoder Representations from Transformers (BERT) [20]. Lee *et al*. used a convolutional neural network (CNN) model, supported by word2vec representations, in the triage phase of genomic variation resources, outperforming the precision of SVM models by up to 3% [21]. This approach increased the precision by up to 1.8 times when compared to query-based triage methods of UniProtKB. Similarly, Burns *et al*. use a combination of CNN and recurrent neural network (RNN) models to scale up the triage of molecular interaction publications [22].

**Figure 1:**
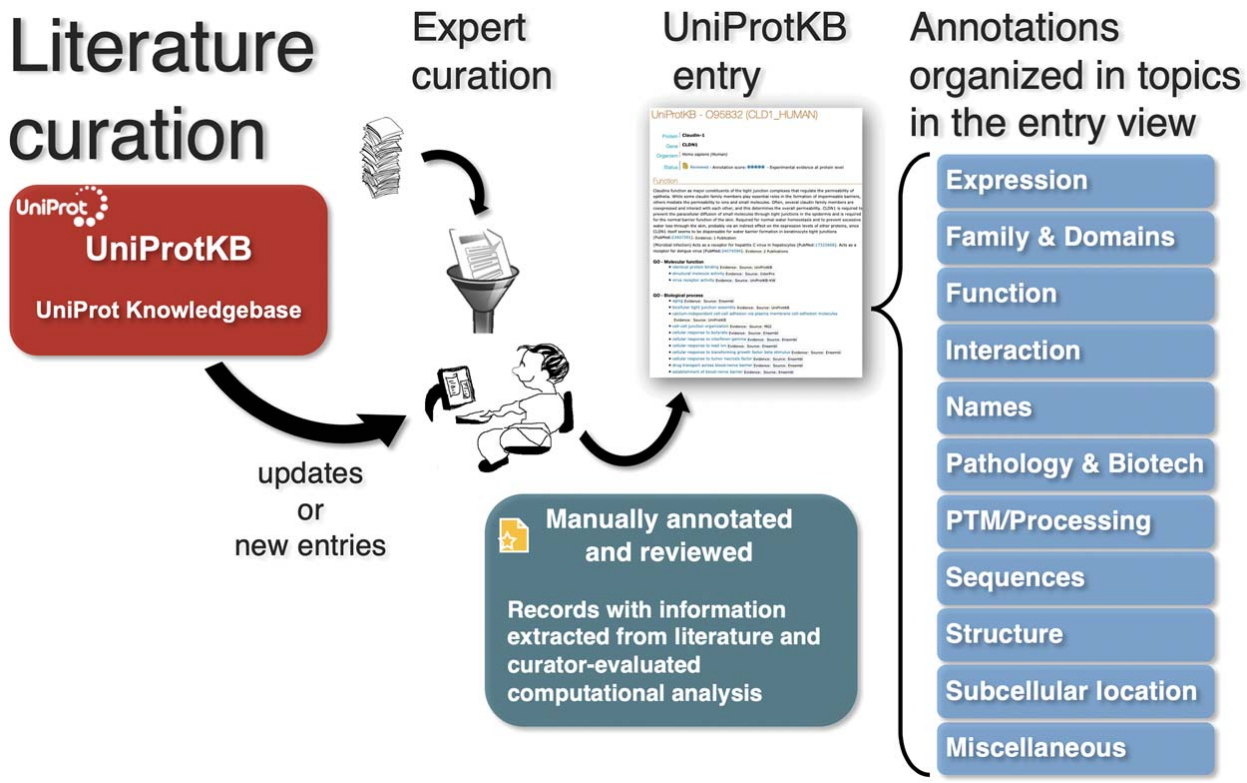
UniProt Knowledgebase annotation process. Manually protein-annotated documents (Swiss-Prot) are associated with UniProt entry categories (Function, Name & Taxonomy, etc.) according to the type of information available in the publication, improving the organization of the annotations within the knowledge base. A much larger set of publications (TrEMBL) is then automatically annotated according to their source characteristics.

To capture the breadth of publications about proteins and make it easily available to users, UniProt compiles additional bibliographies from three types of external sources—databases, community and text mining—which complements the curated literature set with additional publications and adds relevant literature to entries not yet curated [4, 23]. UniProtKB publications in reviewed entries are categorized on 11 pre-defined topics—Expression, Family & Domains, Function, Interaction, Names, Pathology & Biotech, PTM/Processing, Sequences, Structure, Subcellular Location and Miscellaneous—based on the annotation they contribute to the protein entry, e.g. a paper that is the evidence source for a protein’s catalytic activity will be included in the category Function (Figure 1). On the other hand, to automatically categorize additional publications, UniProtKB uses the information from the underlying external sources (usually, databases). For example, the literature provided by the iPTMnet database is under the PTM/Processing category. Nevertheless, this approach is limited as it does not optimally cover all types of evidence available for the specific protein in the publication, other than PTM/Processing in this case. In fact, in publications imported from a number of sources, such as model organism databases, it is not possible to categorize unless the source provides the information.

In contrast to classical text classification problems, it is often the case in biocuration where the same document can be associated with different class sets depending on the biological entity considered. For example, in UniProtKB, the same publication can be categorized into an entry set of the knowledge base for a Protein A and into another entry set for Protein B based on the evidence contained for each protein in the document. Standard document classification models cannot be generalised to this scenario as the input features are the same (i.e. the document) while the output classes are different (i.e. the entry set). To provide a systematic way to categorize the set of additional publications available in UniProtKB, in this paper we propose a multi-label multi-class classification model based on CNN that classifies publication-protein pairs into the 11 UniProtKB entry topics. We use candidate evidence sentences, selected based on availability of protein information, to create different feature sets out of a unique document. These feature sets are then embedded into a continuous word representation space and used as input for a deep neural network-based classifier. We compare the effectiveness of the deep learning-based model with baseline classifiers based on logistic regression and SVM models.

## Materials and methods

To classify publications into the UniProtKB protein entry categories, we developed a text mining pipeline based on a CNN model, so-called UPCLASS. The UPCLASS classification model was trained and evaluated using a large expert curated literature dataset available from UniProtKB. In this section, we describe the methods used to automatically and systematically classify scientific articles in the knowledge base.

### Candidate sentences for annotation evidence

**Figure 2:**
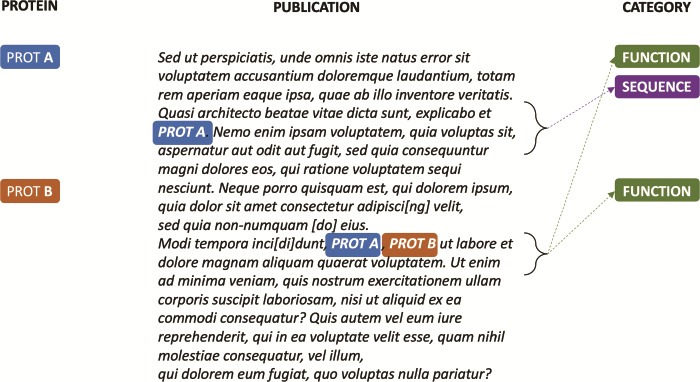
Synthetic annotation example illustrating how a single publication can be associated with different sets of UniProtKB entry categories.

A key challenge for classifying publications according to the UniProtKB entry categories is that, for the same document, a few to thousands of proteins can be annotated with different categories based on the evidence provided in the text. For example, as shown in Figure 2, if for Protein A there is evidence in the article for the Sequence and Function categories, and for Protein B, there is evidence only for the Function category, the publication will be annotated with different class sets for the different protein entries in knowledge base. However, since only a few articles are expert annotated per protein, usually with little redundancy on the type of information they bring to the knowledge base (i.e. an UniProtKB entry category), the protein itself cannot be directly used as a learning feature for a category because it is not an informative feature. In a classical document classification scenario, in which a label set is associated with a single document or to a document–accession pair, the classifier would always receive as input the same set of informative features (i.e. the document) independent of the labels associated with the pair document–accession. Thus, the classifier would not be able to learn the actual classes associated with the document–accession pair, as the triplet “document–accession → categories” tends to appear only once in the knowledge base.

**Figure 3:**
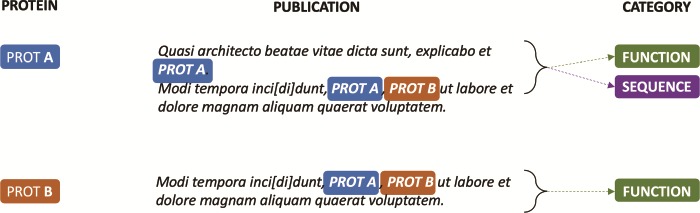
Positive passages extracted from annotations in Figure 2. *k*-nearest (*k* = 0) sentences containing candidate evidence for the protein annotation are concatenated to create a ‘positive’ document. Similarly, sentences that do not contain evidence for the category are concatenated to create a ‘negative’ document (not shown).

To overcome this limitation, we developed a model where a document is divided into ‘positive’ and ‘negative’ sentences, based on whether they provide or do not provide evidence for the protein entry classification. Positive sentences are then concatenated to create a ‘positive’ document for the respective protein annotation. The remaining sentences, i.e. the negative sentences, are similarly concatenated to create a ‘negative’ document. Hence, as shown in Figure 3, different feature sets can be created out of a single document for each document–accession pair and be properly associated with their specific annotation categories.

We hypothesize that the evidence for annotations is provided in the *k*-nearest sentences to the sentence where the protein (or its coding gene) is mentioned. Indeed, as shown by Cejuela *et al*. [14], the *k*−1 sentences accounts for 89% of all unique relationships in the case of protein location evidence. To identify the candidate evidence sentences, occurrence of protein features, such as accession identifier, protein name (recommended, alternative and short), gene name and their synonyms, are searched for in the sentences. For example, for the accession number O95997, we search in the publication sentences for the strings ‘PTTG1_HUMAN’ (accession), ‘Securin’ (recommended name), ‘Esp1-associated protein’ (alternative name), ‘Pituitary tumor-transforming gene 1 protein’ (alternative name), ‘Tumor-transforming protein 1’ (short name), ‘hPTTG’ (short name), ‘PTTG1’ (gene name), ‘EAP1’ (gene synonym), ‘PTTG’ (gene synonym) and ‘TUTR1’ (gene synonym). If at least one match is found, the sentence is added to the positive pool. Subsequent sentences are further concatenated to form the ‘positive’ document for the entry with accession number O95997. These proteins features are available directly from the ‘Names & Taxonomy’ section of the UniProtKB for each accession number.

### Classification model

**Figure 4:**
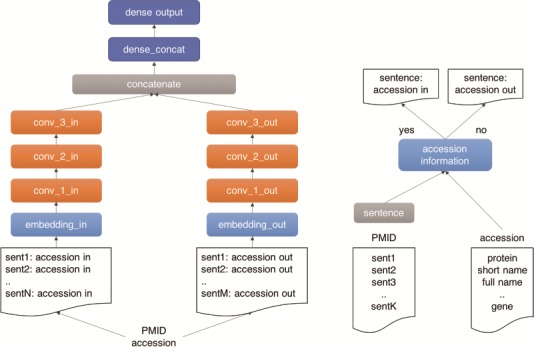
Outline of the UPCLASS CNN-based classification architecture with an embedding layer, three CNN layers followed by two dense layers. The ‘positive’ sentences (accession in) are concatenated and fed to one branch of the model (‘in’ branch). The leftover sentences (accession out) are used to create the ‘negative’ document and fed to the other branch of the model (‘out’ branch).

As illustrated in Figure 4, we use a three-layer CNN architecture for our machine learning classifier. The model has two branches, which receive the positive and negative documents separately. The architecture comprises three main building blocks: (i) an input block (light blue), composed by an embedding layer (width = 1500), which receives the document tokens and the pre-trained word vectors from a paragraph2vec model trained on 10^6^ Medline pre-processed abstracts with protein information; (ii) a CNN block (orange), composed by three CNN layers with 128 channels, kernel width equal to 5 and ReLU activation, followed by a batch normalization layer, which exposes a max pooling output followed by a drop-out layer (drop-out rate of 50%); and (iii) a dense block (dark blue), composed by two dense layers, the first layer (width = 128) receives the concatenated output of the CNN branches as input, followed by an output layer (width = 11) with a Softmax activation function. The outputs of the CNN branches are then concatenated and fed to the dense layer. The model was trained in 50 epochs with early stopping set for five consecutive epochs without improvement in the validation set. The model was implemented using the Keras framework in Python 3.

### Training and test collection

To train our model, we used an expert annotated collection of ~483 k examples available from UniProtKB. In total, the collection contains ~201 k unique manuscripts with an average of 2.4 proteins annotated per article (min = 1, max = 9329). The training collection was divided randomly in 76.2% for the training set (~368 k samples), 11.7% for the validation set (~56 k samples) and 12.1% for the test set (~58 k samples). The division took into account the constraint that a publication should not be split in different sets, as it is commonly the case when a protein is annotated for a category in a publication, other proteins share the same annotation. Full text publications were extracted from PubMed Central when available; otherwise, MEDLINE abstracts were used. In total, 96% of the collection was composed only by abstracts, with 5% of full-text articles in the training set, 6% in the validation set and 8% in the test set. The data used in the experiments is available at https://doi.org/10.5281/zenodo.3672781.

In Table 1, the distribution of examples per category in the training collection is presented. As previously discussed, the number of unique examples labelled per protein accession varies on average from 1.2 for the Names category to 2.2 for the PTM / Processing category. On the other hand, for categories like Names, PTM/Processing and Sequences there is less than one unique document per accession, i.e. it is often the case that a few proteins for these classes are annotated in the same publication. Moreover, we can notice that there is a concentration of samples in some classes, in particular Sequences, which is present in more than 45% of the examples, while Structure, Names and Family & Domains are present in 5% or less.

**Table 1:** Distribution of categories in the manually annotated training collection from UniProtKB

**UniProtKB entry category**	**Examples**	**Unique accessions**	**Unique documents**
Expression	53 274	35 128	34 446
Family & domains	4910	3807	3310
Function	105 417	49 896	72 674
Interaction	60 252	28 318	30 646
Names	11 334	9130	1100
Pathology & biotech	39 870	23 573	32 410
PTM/Processing	69 080	31 142	17 335
Sequences	217 879	130 288	109 333
Structure	25 569	14 257	19 553
Subcellular Location	48 876	31 866	28 793
Miscellaneous	111 454	52 724	16 812
**Total**	**483 159**	**163 913**	**201 358**

Examples: number of document–accession examples annotated with a category in the training collection. Unique accessions: number of unique accessions annotated with a category in the training set. Unique document: number of unique publications annotated with a category in the training set

### Pre-processing and word embeddings

In the pre-processing phase, we pass the training collection and the protein features (name, gene, etc.) through an NLP pipeline. First, sentences are split using a Punkt sentence tokenizer. Then, stopwords are removed, characters are converted to lowercase and non-alphanumerical characters are suppressed. The resulting tokens are stemmed and stem words smaller than two characters are removed. Finally, numerical sequences are replaced by the token _NUMBER_. Table 2 shows an example of the resulting sentences after passing the manuscript through the pre-processing pipeline.

**Table 2:** Resulting sentences after passing through the pre-processing pipeline

**Original text**	**Pre-processed sentences**
YddV from *Escherichia coli* (Ec) is a novel globin-coupled heme-based oxygen sensor protein displaying diguanylate cyclase activity in response to oxygen availability. In this study, we quantified the turnover numbers of the active [Fe(III), 0.066 min(-1); Fe(II)-O(2) and Fe(II)-CO, 0.022 min(-1)] [Fe(III), Fe(III)-protoporphyrin IX complex; Fe(II), Fe(II)-protoporphyrin IX complex] and inactive forms [Fe(II) and Fe(II)-NO, &lt;0.01 min(-1)] of YddV for the first time.	yddv escherichia coli ec novel globin coupl heme base oxygen sensor protein display diguanyl cyclas activ respons oxygen avail studi quantifi turnov number activ fe iii _NUMBER_ min fe ii fe ii co _NUMBER_ min fe iii fe iii protoporphyrin ix complex fe ii fe ii protoporphyrin ix complex inact form fe ii fe ii _NUMBER_ min yddv first time

The word embedding weights were pre-trained in a pararaph2vec to model using the training collection [24]. In addition to adapting to the pre-processed text, the motivation was that locally trained word embeddings provide superior word representation, as shown by Diaz *et al*. [25]. Two paragraph2vec models were trained—DBOW (Distributed Bag of Words) and DMC (Distributed Memory Concatenated)—through 100 epochs with word and document vector size of 200 and window of 10, the optimal values found during the training phase for the classification models. The gensim Python library was used to train the pararaph2vec models.

### Evaluation criteria

Results are reported using standard multi-label classification metrics—Precision, Recall and F1-score—and are compared to a baseline model based on logistic regression. Student’s *t* test is used to compare the classifier models, and results are deemed statistically significant for *P* value < 0.05. As most of the publications contain more than one annotation per protein and they are often classified into the same classes, e.g. a paper containing structure information for several proteins, the predictions might not be independent for each sample. Thus, we report results for real curation use-cases but also consider only one unique random annotation per publication. Finally, it is also important to notice that a system that provides automatic annotations to knowledge bases should aim first at high precision. Nevertheless, in our case, we expect the classifier to go beyond the coverage provided by standard provenance classification, and, hence, demonstrate also high recall.

## Results

Table 3 shows the performance of the classification models used to categorize document–protein accession pairs according to UniProtKB entries. Three classification models were assessed—logistic regression (baseline), SVM and CNN—in two versions: ‘not tagged’ and ‘tagged’. The ‘not tagged’ version of the logistic regression and SVM classifiers received as input a 400-dimensional feature vector created from the concatenated output of the DBOW and DMC paragraph2vec models applied to a pre-processed publication. The ‘tagged’ version received as input an 800-dimensional feature vector, 400 for the positive document and 400 for the negative document, created by tagging protein features against the publication sentences. Similarly, the CNN ‘not tagged’ model received a 1500 token vector per document in the embedding layer while the CNN ‘tagged’ model received a 1500 token vector for each branch of the model (positive and negative). The token weights for the embedding layer of the CNN models were provided by the trained DBOW document embedding model.

**Table 3:** Micro and macro average results for the not tagged and tagged models obtained from the test set of 58k records

**Model**	**Micro**			**Macro**		
	**Precision**	**Recall**	**F1-score**	**Precision**	**Recall**	**F1-score**
**Logistic not tagged**	0.63	0.42	0.50	0.55	0.42	0.50
**Logistic tagged**	0.55	0.66	0.60	0.48	0.60	0.53
**SVM not tagged**	0.74	0.43	0.54	0.56	0.28	0.37
**SVM tagged**	0.75^*^	0.38	0.50	0.64	0.25	0.36
**CNN not tagged**	0.67	0.76^*^	0.71	0.68^*^	0.46	0.55
**CNN tagged**	0.69	0.74	0.72^*^	0.60	0.63^*^	0.62^*^

Highest results are shown in bold. Asterisk denotes statistically significant improvement

Overall, the CNN tagged model achieved the highest performance in terms of the F1-score metrics, outperforming all the other models for both micro and macro averages (*P* < 0.05). It outperformed the baseline logistic regression classifier in absolute values by 12% and by 9% for the micro and macro F1-score metrics, respectively. The SVM tagged model achieved the highest micro precision and the CNN not tagged model achieved the highest macro precision (*P* < 0.05), both at the expense of recall. Similarly, recall performance varies depending on how the results are aggregated. Micro recall is highest for the CNN not tagged model, and macro recall is highest for the CNN tagged model. Since some of the categories had relatively few examples in the training set, the macro average metrics provide better insights on how the models are able to deal with class imbalance, as the macro metrics treat all classes equally, independent of their frequency in the training set. To this end, the CNN tagged model has an outstanding performance, increasing the F1-score metric by 7% when compared to the CNN not tagged model.

As publications are often annotated for several proteins (thousands in some cases), the document–accession pairs are not necessarily independent samples. Thus, we modified the test set to contain only one sample of a document per unique category set. Accessions with the same annotation for a publication were randomly suppressed from the collection. This resulted in a test set of ~26 k records, a reduction of 55% when compared to the original set of ~58 k samples. The results of these tests are shown in Table 4. There was a relevant drop in performance for the CNN models, e.g. 4 and 6% in micro average F1-score for the not tagged and tagged models, respectively, and an overall relative improvement in relation to the CNN models for the logistic and SVM models. Nevertheless, the CNN models still outperform the baseline and SVM when considering the F1-score metrics. The best model in this setting is now the CNN not tagged, with F1-scores of 0.67 and 0.54 for the micro and macro averages, respectively. Thus, for an individual independent classification, the CNN not tagged classifier is likely to provide the best answer while for a collection with the similar distribution to UniProtKB’s categories, the CNN tagged model provides the best results.

**Table 4:** Micro and macro average results for the not tagged and tagged models obtained from the test set of unique document→categories pairs (around 26 k samples)

**Model**	**Micro**			**Macro**		
	**Precision**	**Recall**	**F1-score**	**Precision**	**Recall**	**F1-score**
**Logistic not tagged**	0.56	0.68	0.61	0.45	0.56*	0.50
**Logistic tagged**	0.59	0.66	0.62	0.46	0.53	0.49
**SVM not tagged**	0.73	0.43	0.54	0.62*	0.25	0.36
**SVM tagged**	0.75*	0.45	0.56	0.61	0.27	0.38
**CNN not tagged**	0.64	0.71*	0.67*	0.54	0.54	0.54*
**CNN tagged**	0.65	0.66	0.66	0.55	0.49	0.52

Highest results are shown in bold. Asterisk denotes statistically significant improvement

### Prediction comparison for the not tagged and tagged models

Out of the ~22 k unique publications in the test set, around 7 k (31%) were labelled with two or more category sets. In Table 5, we show three non-exhaustive examples, highlighting the different outcomes for the not tagged and tagged CNN classifiers for such cases. For the first publication, PMID 11847227, five distinct category sets were annotated for the different accessions. As expected, the not tagged model associated only one type of category set to all document–accession pairs while the tagged model changed the predicted classes according to the accession features. This led to an increase in the micro average F1-score from 0.37 for the not tagged to 0.73 for the tagged results. In the second example, PMID 15326186, both models behave similarly, providing only one set of categories, independent of the accession information. This situation is usually seen for the tagged model when the classifier is not able to tag accession information in the document or all sentences in the abstract contain a protein feature token. Thus, a unique set of features, i.e. the publication vector, is used for classification. Finally, in the third example, PMID 10427773, the tagged model has more false positive predictions, lowering its precision when compared to the not tagged model by 2% (F1-score of 0.83 and 0.81, respectively).

**Table 5:** Examples of prediction output for the CNN not tagged and tagged models

**PMID**	**Accession**	**Gold standard**	**Prediction**	
			**Not tagged**	**Tagged**
11 847 227	Q9BTW9	Function	Function, pathology & biotech, sequences	Function
11 847 227	O75695	Function, interaction, miscellaneous	Function, pathology & biotech, sequences	Function, interaction
11 847 227	Q15814	Function, pathology & biotech	Function, pathology & biotech, sequences	Function
11 847 227	Q9Y2Y0	Interaction	Function, pathology & biotech, sequences	Function, interaction
11 847 227	P36405	Interaction, pathology & biotech	Function, pathology & biotech, sequences	Function, interaction
15 326 186	A7E3N7	Expression	Expression, function	Expression, function
15 326 186	Q8NFA2	Expression, function	Expression, function	Expression, function
15 326 186	Q672J9	Expression, function, sequences	Expression, function	Expression, function
15 326 186	Q672K1	Expression, sequences	Expression, function	Expression, function
15 326 186	Q8CJ00	Function	Expression, function	Expression, function
10 427 773	Q9SAA2	Expression	Expression, sequences	Expression, sequences
10 427 773	Q9SXJ6	Expression, sequences	Expression, sequences	Expression, sequences
10 427 773	Q9S834	Expression, sequences	Expression, sequences	Expression, sequences, subcellular location
10 427 773	Q9XJ36	Expression, sequences	Expression, sequences	Expression, sequences
10 427 773	Q9SXJ7	Expression, sequences, subcellular location	Expression, sequences	Expression, sequences
10 427 773	Q9XJ35	Expression, sequences, subcellular location	Expression, sequences	Expression, subcellular location
10 427 773	P42762	Expression, subcellular location	Expression, sequences	Expression, sequences
10 427 773	P62126	Sequences	Expression, sequences	Expression, sequences

While the CNN not tagged model provides only one type of output independent ofthe accession evidence in the publication, the tagged model attempts to predict the categories according to the accession features. For brevity, only unique examples of document→categories pairs are shown

### Classifier performance analyses

**Figure 5:**
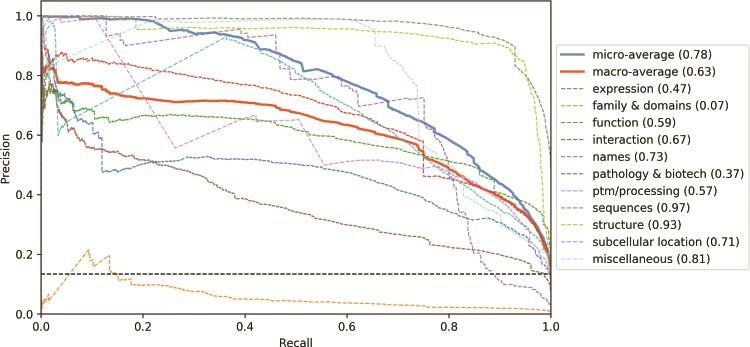
Precision-recall curves for the UniProtKB categories obtained from the CNN tagged classification. Mean average precision is shown in parentheses. Black horizontal dashed line: performance of a random classifier.

As shown in Figure 5, to understand the impact of the different categories in the classifier’s performance, we analysed the precision-recall curve for the CNN tagged model (other classifiers show similar curves). Three categories have mean average precision (MAP) above 75%: Sequences, Structure and Miscellaneous. On the other hand, three categories have MAP lower than 50%: Expression, Family & Domains and Pathology & Biotech. The correlation between the number of examples in the training set and the category performance is moderate (*ρ* = 0.53). Indeed, the Structure category, for example, is only present in 5% of the training examples, however it has one of the highest MAP. Differently, the Expression category is present in 11% of the examples (the median value among the categories) but has one of the lowest MAP. The Family & Domains category, which has only 1% of the training set records, was not learned by the classifier (MAP = 0.07). However, the Names category has 2% of the examples and a MAP 10 times higher (MAP = 0.73). Hence, the class imbalance alone is not enough to explain the difference in performance among the categories.

**Figure 6:**
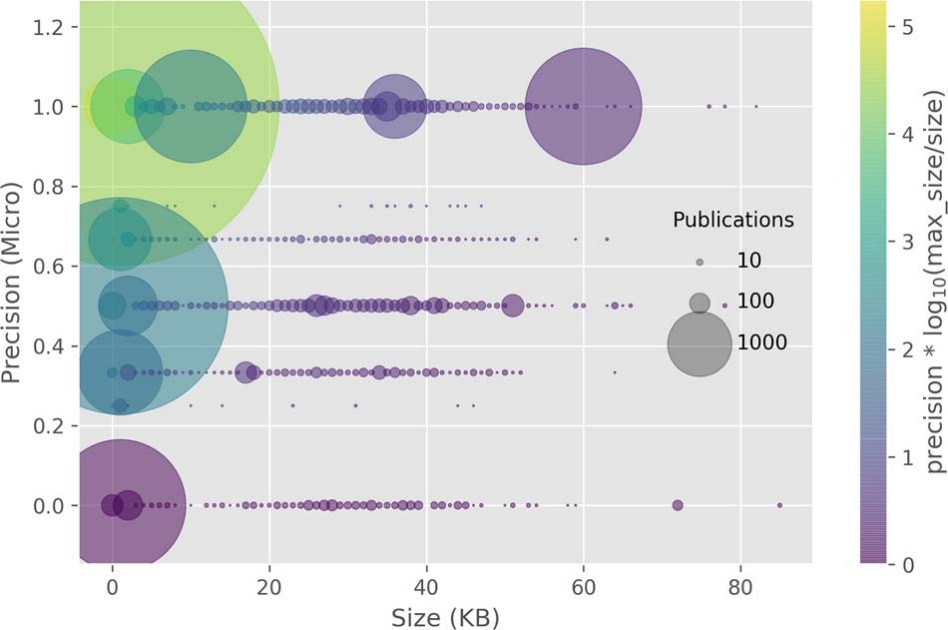
Classifier precision as a function of the publication size. There is no correlation between the size of the input size and precision. Circle size: number of publications within a size bin. Yellow points: high precision and lower ratio between publication size and the max publication size in the test set. Purple points: low precision and higher ratio between the publication size and the max publication size in the test set.

In Figure 6, the classifier prediction as a result of the input size is shown. Documents with size around 4 k bytes contained only the abstract section and composed more than 95% of the test set. The remaining 5% were composed of full-text documents or those with at least some extra sections in addition to abstract, such as figure and table captions. The correlation of classifier performance and size of the publication, or by proxy, between the type of annotated publication: abstract or full text, is very weak (*ρ* = 0.09). Thus, it seems that most of the evidence for the categories is provided in the abstract, apart from the Expression, Family & Domains and Pathology & Biotech categories, which have a MAP lower than 0.5.

**Figure 7:**
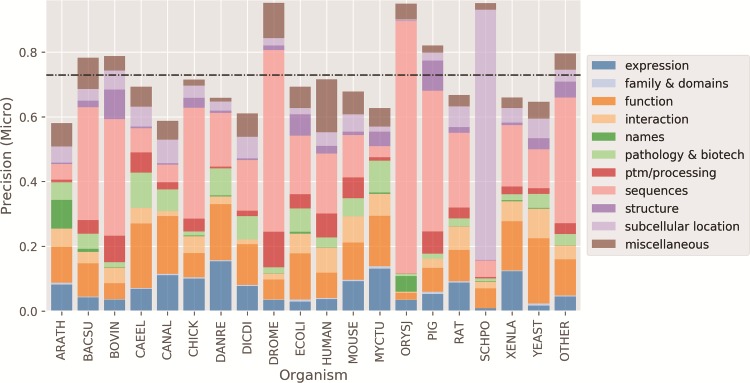
Micro average precision performance per organism. Higher precision for organisms happens when there is a concentration of categories. Black dashed horizontal line: mean organism precision.

Finally, in Figure 7 we show the micro average precision results of the CNN tagged classifier for the 19 most common organisms in the test set. For the organisms with higher precision, *Drosophila melanogaster* (DROME), Schizosaccharomyces pombe (SCHPO) and *Oryza sativa subsp. japonica* (ORYSJ), the majority of the protein entry annotations belong to a few classes (median frequency of classes of <2%). These organisms were mostly annotated for Sequences and Subcellular Location categories (~60% or more of the annotations), which had overall high to moderate-to-high precision performance, respectively, as shown in Figure 5. Conversely, organisms with well-distributed annotations among the 11 categories in the test set (median distribution of ~9%) had the poorest precision scores: *Arabidopsis thaliana* (ARATH), *Candida albicans* (CANAL) and Dictyostelium discoideum (DICDI). The low-performing categories are likely to have impacted negatively the precision of these organisms.

### UniProtKB category evidence annotation

To measure the performance of the tagging method for detecting candidate evidence sentences, we compared sentences from 20 manually annotated abstracts with the positive and negative sentences created by our naïve string-matching algorithm. In total, 61 sentences distributed among 7 categories—Expression, Function, Interaction, Pathology & Biotech, PTM/Processing, Sequence and Subcellular Location—were tagged as positive. The algorithm achieved micro precision of 0.42 (28 out of 67 sentences) and micro recall of 0.46 (28 out of 61 sentences). For 4 abstracts (20%), no positive sentence was detected, i.e. none of the protein descriptors was found in the abstract using the string matching method. In this case, the whole abstract was considered as positive. For the other 5 articles (25%), no true positive sentences were tagged. After assessing the classification performance for this test set (F1-score of 0.70), there was no correlation between the detection of candidate sentences and the prediction of the individual document-accession pairs (*ρ* = 0.02).

## Discussions

We investigated the use of a supervised CNN classifier to automatically assign categories to document-accession pairs curated in the UniProtKB to help scale up publication categorization into the knowledge base entry categories. The classifier was trained and evaluated using a collection of 483 k document-accession pairs annotated by biocurator experts. To overcome the issue of multiple category sets associated with a single publication, we proposed an effective strategy to tag publication sentences with protein features and create different feature sets out of a single document entry. Results showed statistically significant improvements upon models that use only the publication as a classification feature, improving the F1-score up to 12% (micro) when compared to the logistic regression baseline and up to 7% (macro) when compared to the not tagged CNN version.

While for some database resources it has been shown that expert curation can keep up with the exponential growth of the scientific literature [4], scaling-up biocuration remains a challenge. A key success factor for UniProtKB’s scalability, for example, is that the set of expert curated literature in the knowledge base focuses on non-redundant annotations for proteins. It relies on external sources for the contribution of additional literature and on UPCLASS for its classification. Indeed, the implementation of UPCLASS has enabled the classification of more than 30 million document–accession pairs according to the entry categories, which were previously displayed as unclassified in UniProtKB [3]. UPCLASS is publicly available as a web service through the URL address: https://goldorak.hesge.ch/bioexpclass/upclass/ and its code is maintained at https://github.com/dhteodoro/upclass.

In addition to direct classification, models as provided by UPCLASS could be used in other automatic phases of the biocuration process, such as document triage, helping curators to reduce the search scope. In the context of UniProtKB curation workflow, it could be used to prioritize UniProtKB entries that are unreviewed proteins with publications in the additional bibliography belonging to some category of interest. Furthermore, UPCLASS could be used to update reviewed entries lacking a Function annotation, for example, but for which there are papers in the additional bibliography containing functional annotation. In [10], UPCLASS was used in a question–answer model to classify the type of search questions and their respective result sets in biomedical metadata repositories, e.g. search for gene expression data or search for protein sequencing data. While this approach did not lead to improvements in information retrieval performance of datasets, further investigation is needed, in particular in a context where the type of classification material is aligned with the training set type. Overall, given the outstanding results provided by the CNN models when compared to the baseline model but even to more powerful frameworks, such as SVM, we expect that such models could be extended to other literature curation domains, for example, in prior art search and classification of patents [26, 27].

Recent advances in deep learning for textual data fuelled by recurrent models (LSTM, GRU, etc.) and contextual embedding (e.g. ELMo, BERT) have demonstrated state-of-the-art performance in several downstream tasks. In this work, however, we opted for a model with lower algorithm complexity, i.e. word2vec-based embeddings combined with a CNN model, as our main classifier due to the scale of the production environment found in UniProtKB, where samples in the order of O(10^8^) need to be encoded in a lower-dimensional space and classified. As shown in several works [28, 29], the performance of classification models varies significantly according to the real-world tasks. Thus, by using models that are simpler algorithmic-wise but yet are high performing, we achieved a good compromise between processing time and precision.

### Classification performance per category, document size and organism

We analysed the performance of the CNN tagged classifier according to three dimensions: category, document size and annotated organism. Despite the good performance of the classification models, there is still room for improving precision for some of the UniProtKB classes. Several attempts were made to increase UPCLASS outcomes using imbalanced learning methods, such as under sampling and Tomek links [30]. However, they did not lead to overall improvements. An alternative would be to use a classifier ensemble, as proposed in [15]. However, this approach is too expensive for deep learning models due to the learning cost. We also tried to change the number of *k*-nearest sentences in the tagged models, but it did not lead to positive changes in performance either. It would be interesting to explore the addition of expert categorization rules, in particular of UniProtKB mapping rules, as a strategy to increase performance while keeping the training complexity relatively low. The analyses of the classification precision according to the size (or type, i.e. abstract vs. full text) of the document shows that there is no correlation between these two dimensions. While it might be counter-intuitive, several works have demonstrated that for classification tasks, the performance of abstracts is at least equivalent to full texts, if not better [31]. Yet, it could be relevant to explore the use of different classifiers for abstracts and for full texts. Finally, performance analyses according to annotated organism show that better prediction outcomes are mostly a result of high-performing class annotations being concentrated on some organisms. Thus, it seems that the classification precision for organisms is not related to the way they are curated.

### Correlation between correctly tagged sentences and classification results

We used a collection of 20 manually annotated documents at the sentence level to assess the performance of the string-matching method based on protein features to tag evidence sentences. When measuring the impact on the classifier’s performance, results show that there is no correlation between correctly tagged sentences and correctly predicted categories. We have two hypotheses for this lack of correlation. First, we believe that just splitting the sentences that contain accession information would be sufficient for the classifier to more effectively learn the category features. Even if the sentences did not have exactly the evidence used by biocurators, the most important would be to recall sentences with protein information. Indeed, while the correlation of the classifier’s precision with the tagging method precision is only 0.02, the correlation between the classifier’s precision and the number of positive tagged sentences is 0.26. Notice that there is only a marginal improvement from the not tagged to tagged model if we consider micro average F1-scores (0.71 to 0.72). Thus, the recall increase in positive sentences would provide the edge for the tagged model. The second and most straightforward hypothesis is that the lack of correlation is related to the small size and statistical power of the annotated set. To investigate both assumptions, a larger annotated set would be needed, and it is out of the scope of this paper.

### Limitations

Abstracts compose the majority of the collection used in the experiments. Nevertheless, a large body of the annotation evidence is expected to be found in the methods and results sections of full-text articles. The logistic regression and SVM methods use the whole document albeit compressed in a 200-vector, which was identified as the best value during the training phase. On the other hand, for the CNN models the documents were truncated in 1500 tokens (1500 for each branch in the tagged model) due to performance reasons, which limits the comparison with the baselines.

In UniProtKB, a single article can be annotated for many proteins, sometimes with the same class; hence, the prediction results are not independent, biasing the assessment. We tried to mitigate this issue by creating a test set with unique ‘document → categories’ pairs; however, examples in this set still cannot be considered as fully independent, as one document can be associated with class sub-sets, e.g. PMID → [Function] and PMID → [Function, Sequences].

To detect annotation evidence, we used a naïve approach based on string matching against a set of protein descriptors, including name and gene, to improve algorithm complexity. Nevertheless, more sophisticated NER models could be used instead to detect protein mentions, for example character-level neural network as proposed by Gridach [32]. In particular, our approach could mismatch some protein names, including numbered proteins, such as Il-2 and Il-3, depending on how they are written. Moreover, by removing tokens smaller than two characters in the pre-processing phase, some protein mentions might have been excluded from the text, reducing protein/gene matching and thus potential evidence recall.

## Conclusion

To provide a systematic way of categorizing computationally mapped publications in UniProtKB, in this paper we investigated the use of a supervised CNN classifier for assigning categories to pairs of document–protein accessions. To overcome the issue of multiple category sets associated with a single publication, we proposed an effective strategy to tag publication sentences with protein features and create different feature sets out of a single document entry, which were then fed to different CNN layers. Results showed statistically significant improvements upon models that use only the publication as classification features, improving the F1-score up to 22 and 12% when compared to a logistic regression baseline for the micro and macro averages, respectively. Moreover, our results show that text classification supported by CNN models provided an effective way to classify publications according to the UniProtKB entry categories. As future work, we will investigate whether the classification performance for underperforming classes can be improved by adding expert knowledge into the model. Furthermore, we want to explore whether sentences used as evidence for categorization can be relocated automatically from the classifier model.
